# Environmental Antecedents of Foodborne Illness Outbreaks, United States, 2017–2019

**DOI:** 10.1016/j.jfp.2024.100293

**Published:** 2024-05-06

**Authors:** Meghan M. Holst, Sabrina Salinas, Waimon T. Tellier, Beth C. Wittry

**Affiliations:** 1 Centers for Disease Control and Prevention, National Center for Environmental Health, 4770 Buford Highway, Atlanta, GA 30341, USA; 2 Harris County Public Health, Environmental Public Health, 1111 Fannin Street, Houston 77002, USA; 3 Washington State Department of Health, Office of Communicable Disease Epidemiology, 1610 NE 150th Street, Shoreline, WA 98155, USA

**Keywords:** Environmental antecedents, Environmental assessment, Environmental health, Foodborne illness outbreak, Illness prevention, Root cause

## Abstract

Foodborne outbreak investigations often provide data for public health officials to determine how the environment contributed to the outbreak and on how to prevent future outbreaks. State and local health departments are responsible for investigating foodborne illness outbreaks in their jurisdictions and reporting the data to national-level surveillance systems, including information from the environmental assessment. This assessment is designed to describe how the environment contributed to the outbreak and identifies factors that contributed to the outbreak and environmental antecedents to the outbreak. Environmental antecedents, also referred to as root causes, are specific reasons that allow biological or chemical agents to contaminate, survive, or grow in food. From 2017 to 2019, 24 jurisdictions reported 1,430 antecedents from 393 outbreaks to the National Environmental Assessment Reporting System. The most reported antecedents were lack of oversight of employees/enforcement of policies (89.1%), lack of training of employees on specific processes (74.0%), and lack of a food safety culture/attitude towards food safety (57.5%). These findings highlight the critical role that employees play in restaurant food safety and are heavily influenced by restaurant management, who can exercise active managerial control to manage these antecedents. Identifying antecedents during investigations is essential for understanding the outbreak’s root cause and implementing sustainable corrective actions to stop the immediate outbreak and future outbreaks.

The Centers for Disease Control and Prevention (CDC) estimates that around 48 million foodborne illnesses occur each year in the United States. This number includes 128,000 hospitalizations and 3,000 deaths (Scallan et al., 2011). Illnesses associated with outbreaks account for a small portion of the annual foodborne illness incidence (Dewey-Mattia et al., 2018). However, data from foodborne outbreak investigations can provide detailed information that helps determine how the environment contributes to outbreaks. That information can be used to prevent future outbreaks (Dewey-Mattia et al., 2018). The lessons we learn from outbreak investigations can also be used to prevent sporadic cases of foodborne illnesses.

State and local health departments are responsible for investigating foodborne illness outbreaks and reporting the data to national-level surveillance systems managed by the CDC. CDC aggregates these data to detect national trends and identify systematic food safety issues. The National Environmental Assessment Reporting System (NEARS) is a CDC surveillance system through which some state and local health departments voluntarily report data from their investigations of foodborne illness outbreaks at retail food establishments (Centers for Disease Control and Prevention, 2022b). Specifically, they report data from the environmental health component of the investigation, typically called the environmental assessment. The assessment is designed to describe how the outbreak environment contributed to the introduction or transmission of the agent that caused the outbreak (Centers for Disease Control and Prevention, 2022c). NEARS collects two key sets of environmental assessment data about the outbreak (Freeland et al., 2019; Moritz et al., 2023).

The first set is outbreak contributing factors—the practices that most likely led to contamination, proliferation, or survival of agents in the environment (Centers for Disease Control and Prevention, 2023). The second set is environmental antecedents, hereafter referred to as antecedents, of the outbreak. Antecedents are the circumstances and situations that led to the contributing factors. For example, an investigation may identify the contributing factor to a norovirus outbreak as contamination of food by an ill employee, and the antecedent as insufficient staffing, which led to the employee not being allowed to stay home while ill.

Identifying the contributing factors and antecedents is essential to understanding the outbreak’s root cause and implementing sustainable corrective actions to stop the outbreak and future outbreaks (Firestone et al., 2018). Collecting and analyzing these data at a national level are essential for informing efforts to prevent future outbreaks in retail environments across the country. The data can be used to inform national food safety policy and training focused on reducing contributing factors and antecedents (Centers for Disease Control and Prevention, 2022a; Matis et al., 2017; Wittry et al., 2022). To contribute to these goals, the current study presents data on the contributing factors and antecedents for outbreaks reported to NEARS from 2017 to 2019.

## Materials and Methods

Between 2017 and 2019, state and local health departments reported data to NEARS from 799 outbreak investigations with a suspected or confirmed foodborne agent. CDC defines a foodborne illness outbreak as an incident in which two or more people experience a similar illness resulting from the ingestion of a common food (Centers for Disease Control and Prevention, 2015). The dataset for this paper is limited to the 393 outbreaks that had at least one reported antecedent. The outbreaks occurred in Alaska, California, Connecticut, Delaware, Fairfax County (VA), Georgia, Harris County (TX), Indiana, Iowa, Jefferson County (CO), Maricopa County (AZ), Massachusetts, Michigan, Minnesota, New York City, New York State, North Carolina, Oregon, Rhode Island, South Carolina, the Southern Nevada Health District, Tennessee, Washington, and Wisconsin.

Investigators conducting environmental assessments for outbreaks in retail food establishments engage in a variety of data collection activities. These include kitchen observations, reviews of records, and staff interviews. Data from these activities, along with data obtained from epidemiological and laboratory investigations, are reported to NEARS (Lipcsei et al., 2019; Moritz et al., 2023). Additionally, investigators report any contributing factors or antecedents they identified during their outbreak investigation. The identification is based on their review of the totality of the data collected during the observation and their professional judgment and experience. Investigators select contributing factors from a predetermined list of 32 contributing factors. The list of factors is derived from an analysis of historical data from outbreak investigations (Bryan, 1978; Weingold et al., 1994). The investigators also select antecedents from a list of 17 potential antecedents and an option to write in an ‘other’ response. This list was developed by food safety experts using the food system environmental antecedent conceptual model (Selman & Guzewich, 2014). The model theorizes that there are five main groups of environmental antecedents that influence food safety in establishments. The groups are *people* (behaviors, characteristics, and attitudes of people working in the establishments); *processes* (characteristics of the processes used to prepare food and food preparation complexity); *economics* (costs and profit margins); *equipment* (the physical layout and equipment of establishments); and *food* (the inherent qualities of food prepared in establishments). Investigators can report multiple contributing factors and antecedents for each outbreak.

We created the dataset for this study with SAS 9.3 statistical software (SAS Institute, Cary, NC), then used Microsoft Excel to conduct descriptive analyses on the data. The analyses focused on outbreak etiologic agents, contributing factors, and antecedents. Additionally, to better understand how outbreak antecedents vary by etiologic agent and contributing factor, we disaggregated the antecedent data by the most common etiologic agents and contributing factors. We also disaggregated the antecedent data on the most common outbreak etiologic agents, contributing factors, and year.

## Results

### Outbreak characteristics.

Of the 393 foodborne outbreaks included in this analysis, 79.1% (311) had an identified agent. Over 70% (72.7% [226]) of these outbreaks with an identified agent had a confirmed etiologic agent and the remaining outbreaks (27.3% [85]) had a suspected etiologic agent. The most common agents were norovirus (39.7%), *Salmonella* (13.5%), and *Clostridium perfringens* (6.4%) (Table 1). Investigations did not identify an etiologic agent in 20.9% (82) of outbreaks. The most commonly identified contributing factors fell into the contamination category: other mode of contamination by a suspected infectious worker (24.4%), cross-contamination of ingredients (14.2%), and bare-hand contact by a suspected infectious worker (14.2%) (Table 2).

### Outbreak antecedents.

Investigators identified 1,430 antecedents associated with the 393 outbreaks. The majority of identified antecedents fell into the people category (67.4%), followed by the process (13.4%), equipment (8.7%), economics (5.9%), other (i.e., antecedents that do not fall into existing categories) (3.3%), and food (1.3%) categories (Table 3).

The top three antecedents fell into the people category: lack of oversight of employees (89.1%), lack of training of employees on specific processes (74.0%), and lack of food safety (57.5%) (Table 3). The next two most common antecedents fell into the process category: staff not following the facility’s processes (24.9%) and insufficient process to mitigate hazard (23.9%).

### Outbreak antecedents by etiologic agent.

The three most common etiologic agents were the virus norovirus, and the bacteria *Salmonella* and *Clostridium perfringens*. The three most common antecedents for all three types of outbreaks were people antecedents: lack of oversight of employees (78.8%, 60.4%, 48.0%, respectively), lack of training of employees (51.3%, 54.7%, and 56.0%, respectively), and lack of food safety culture (39.1%, 52.8%, and 36.0%, respectively) (Table 4). However, the antecedent of lack of oversight of employees was identified far more often for norovirus outbreaks than for the bacterial outbreaks (78.8% vs 60.4%, 48.0%). Additionally, lack of food safety culture was identified more often for *Salmonella* than for *Clostridium perfringens* (52.8% vs. 36.0%).

All three types of outbreaks had equipment antecedents identified, but again, the proportion differed by etiologic agent. More often, the two types of bacterial outbreaks, compared to norovirus outbreaks, had improperly used equipment identified as an antecedent (9.4% and 12.0% vs. 1.3%) (Table 4). Similarly, more often *Clostridium perfringens* outbreaks, compared to norovirus outbreaks, had insufficient equipment capacity identified as an antecedent (28.0% vs. 3.2%).

Lack of sick leave was identified as an antecedent for all three types of outbreaks but was more often identified for norovirus outbreaks than for the two types of bacterial outbreaks (26.3% vs. 9.4% and 4.0%) (Table 4). On the other hand, insufficient process to mitigate hazards was identified more often for the two types of bacterial outbreaks than for norovirus outbreaks (22.6% and 36.0% vs. 8.3%).

### Outbreak antecedents by contributing factors.

The three most common contributing factors fell into the contamination category—other mode of contamination by a suspected infectious worker, cross-contamination of ingredients, and bare-hand contact by a suspected infectious worker (Table 5). The three most common antecedents for the top three contributing factors were the people antecedents of lack of oversight of employees (79.2%, 71.4%, 76.8%, respectively), lack of training of employees (51.0%, 62.5%, and 66.1%), and lack of food safety culture (31.3%, 55.4%, 53.6%). However, the two antecedents of lack of employee training and lack of food safety culture were identified more often for the contributing factors of cross-contamination of ingredients and bare-hand contact by a suspected infectious worker than for other mode of contamination by a suspected infectious worker (62.5%, 66.1% vs. 51.0%; 55.4% and 53.6% vs. 31.3%).

The equipment antecedent of poor facility layout was identified more often for cross-contamination of ingredients than for the two contributing factors associated with suspected infectious workers (12.5% vs. 4.2% and 1.8%) (Table 5). On the other hand, the economics antecedent of lack of sick leave was more often identified for the two contributing factors of suspected infectious worker than for cross- contamination of ingredients (30.2% and 25.0%, vs. 5.4%). Similarly, the people antecedent of low or insufficient staffing was identified more often for bare-hand contact by a suspected infectious worker than for cross contamination of ingredients (14.3% vs. 5.4%).

### Variation by year.

Norovirus and *Clostridium perfringens* outbreaks slightly decreased over time (54.5%, 48.7%, and 48.7%; 9.1%, 8.4%, and 7.0%), while *Salmonella* outbreaks increased slightly (15.6%, 17.6%, and 17.4%) (Table 6). The contributing factors of other mode of contamination by a suspected infectious worker and bare-hand contact by a suspected infectious worker decreased over time (33.0%, 22.4%, and 21.6%; and 20.5%, 13.8%, and 11.1%). Lastly, the antecedent of lack of employee oversight increased over time (85.2%, 81.6%, and 98.7%).

## Discussion

### People, lack of oversight.

The three most common outbreak antecedents identified by investigators were in the people category: lack of oversight of employees, lack of training of employees, and lack of food safety culture. Lack of a food safety culture is defined as the shared values, beliefs and norms that affect mind-set and behavior toward food safety in an organization (Yiannis, 2009). These findings highlight the critical role that employees play in restaurant food safety. The most common antecedents were observed in norovirus, *Salmonella*, and *Clostridium perfringens* outbreaks, indicating that these antecedents support both viral and bacterial outbreaks. However, the antecedent of lack of oversight of employees was identified much more often (> 25 percentage points) for norovirus outbreaks than for the two types of bacterial outbreaks. This suggests a particularly strong association between lack of oversight of employees and norovirus outbreaks. Foodborne norovirus outbreaks are more often associated with ill employees contaminating food than are bacterial outbreaks (Hall et al., 2012). Indeed, most foodborne norovirus outbreaks are associated with ill employees (Hall et al., 2012). Restaurant managers are responsible for preventing employees who are ill with foodborne illness symptoms from working (Food and Drug Administration, 2022). Our data suggest that managerial failure to exclude ill employees from working is a key antecedent to norovirus outbreaks.

### People, lack of sick leave.

Similarly, lack of sick leave for employees was identified as an antecedent more often for norovirus outbreaks (> 18 percentage points) than for *Salmonella* and *Clostridium perfringens* outbreaks. This antecedent was also identified more often for the two suspected infectious worker contributing factors. Given that ill employees are more often associated with norovirus outbreaks than with bacterial outbreaks, lack of sick leave as an antecedent to norovirus outbreaks and to contributing factors related to ill employees is not surprising. Ill employees may not feel they can lose pay; thus, they work while ill and transmit their illness to customers through the food they contaminate. This finding is supported by other research showing that financial issues, such as lack of sick leave and loss of shifts, are cited by employees as reasons for working while ill (Carpenter et al., 2013; Sumner et al., 2011; Norton et al., 2015). Research also shows that mandated paid sick leave reduced instances of employees working while ill (Schneider, 2020). Restaurants can take other steps to manage ill employees. For example, they can create written policies, address reasons why employees work while ill, and create schedules for when an employee is ill (Centers for Disease Control and Prevention, 2022d).

### Active managerial control.

The top three antecedents (lack of oversight of employees, lack of training of employees, lack of food safety culture) and other people antecedents, such as lack of sick leave, are heavily influenced by retail food establishment managers. Active managerial control is the purposeful incorporation of specific actions or procedures by industry management into the operation of their business to attain control over foodborne illness risk factors. Active managerial control can be used to encourage proper food safety practices (Food and Drug Administration, 2022). Some state and local health departments provide an active managerial control toolkit or resource center to assist food establishments in promoting and implementing this concept in their operations (Alexander-Leeder & Gzebb, 2023; Maricopa County Department of Public Health, 2023). State and local health departments have also encouraged food establishments to develop and adopt active managerial control in their operations through incentive programs. Regulators indicated this innovative strategy has been successful as they observed fewer violations in establishments resulting in fewer required inspections by the health department (Retail Food Safety Regulatory Association Collaborative, 2023).

### Equipment.

We found that equipment-related antecedents were more common for bacterial outbreaks than norovirus outbreaks. Restaurants typically rely on equipment for temperature control (e.g., walk-in coolers for cold holding, bain-maries for hot holding) (McCabe-Sellers & Beattie, 2004). When this equipment fails or is used improperly, bacterial pathogens survive and proliferate in food. For example, Wittry et al. found that a common cause of *Clostridium perfringens* outbreaks is the use of hot-holding equipment that is not large enough for the establishment’s operational demand (Wittry et al., 2022). Our finding highlights the importance of using the proper type of equipment and using the equipment properly to prevent bacterial outbreaks.

### Poor facility layout.

The antecedent of poor facility layout was identified more often with the contributing factor of cross-contamination than with contributing factors related to ill employees. Poor facility layout, possibly due to a lack of space or poor design, can support ingredient cross-contamination. For example, a meat preparation area immediately adjacent to a salad preparation area could lead to raw meat juices contaminating the salad preparation area and ingredients. To prevent ingredient cross-contamination due to poor facility layout, the Food and Drug Administration (FDA) recommends that restaurants systematically identify their risks and develop plans for mitigating those risks (Food and Drug Administration, 2022). Ideally, these risks are identified during a construction plan review using hazard analysis critical control point (HACCP) principles as a tool to design safe facility layouts (Conference for Food Protection, 2016). HACCP plans are recommended by the FDA, endorsed by most state and local health departments, and are one of the best ways to mitigate risks in a food establishment (Center for Food Safety and Applied Nutrition, 2006).

### Variations by year.

There are some variations of etiologic agents, contributing factors, and antecedents by year, which could be explained by advanced laboratory testing methods and a push for increased food safety. Norovirus outbreaks decreased while *Salmonella* outbreaks slightly increased. This change could be due to a wider use of whole genome sequencing (WGS). WGS provides a faster, more accurate detection of bacteria (e.g., *Salmonella*), which may have previously been labeled as unknown (Centers for Disease Control and Prevention, 2022e; Rounds, et al., 2020). Contributing factors related to ill food workers decreased from 2017 to 2019. The FDA Risk Factor Study data from 1998 to 2009 showed that compliance for no bare-hand contact with ready-to-eat foods increased over the 10-year study (Food and Drug Administration, 2010), which are actions that prevent food contamination. While this study shows similar results, more research is needed to determine what drives the decrease in bare-hand contact with ready-to-eat foods. Over the past decade, CDC and FDA research findings have focused on improving these food safety practices to prevent norovirus contamination in retail food establishments (Kambhampati et al., 2016; Hoover et al., 2020; Centers for Disease Control and Prevention, 2019).

### Limitations.

There are a few limitations to this study. NEARS is a voluntary reporting system and although the reporting sites are geographically diverse, the data may not be representative of all foodborne outbreaks. The number of outbreaks at each site is likely underreported because health departments do not detect or investigate all outbreaks. Another limitation is the variability in investigation practices across health departments due to different jurisdictional policies and trainings. Identification of antecedents is based on the investigator’s judgment and these judgments may vary.

The findings from this study provide valuable and novel information about antecedents to foodborne outbreaks. The most common antecedents to foodborne illness outbreaks we identified (lack of employee training, lack of employee oversight, and lack of food safety culture) are heavily influenced by restaurant management, who can exercise active managerial control to mitigate these antecedents. Establishment management can work to implement active managerial control in their establishments to help ensure effective oversight and training for employees. There are many resources, from FDA and various health departments across the United States, available to health departments to assist establishments in exercising active managerial control (Food and Drug Administration, 2022; Alexander-Leeder & Gzebb, 2023; Maricopa County Department of Public Health, 2023). Interventions to address common root causes of outbreaks could focus on people as root causes and managerial leadership to improve food safety in an establishment (Kramer et al., 2023; Lee et al., 2021). Identifying antecedents during investigations is essential for understanding the outbreak’s root cause and implementing sustainable corrective actions to stop the immediate outbreak and future outbreaks. Health departments are encouraged to conduct a thorough environmental assessment and identify the root cause of outbreaks to prevent future outbreaks.

## Figures and Tables

**Table 1.** Foodborne outbreak etiologic agents—National Environmental Assessment Reporting System, 2017 - 2019

Agent^a^	*n*	% of outbreaks^b^ (*n* = 393)
**Virus**		
Norovirus	156	39.7
Hepatitis A	2	0.5
**Bacteria**		
*Salmonella* species	53	13.5
*Vibrio* species	10	2.5
*Clostridium perfringens*	25	6.4
*Campylobacter* species	14	3.6
*Escherichia coli*, O157: H7	5	1.3
*Escherichia coli*, other Shiga toxin–producing or verotoxin-producing	7	1.8
*Shigella* species	5	1.3
*Bacillus cereus*	7	1.8
*Staphylococcus aureus*	2	0.5
**Parasite** – *Cyclospora cayetanensis*	8	2.0
**Toxic agent**	9	2.3
**Chemical agent**	2	0.5
**Other agent**	7	1.8
**Unknown agent**	82	20.9
**Total**	394	100.4

a More than one agent can be reported per outbreak.

b The denominator is the number of outbreaks that identified an environmental antecedent.

**Table 2:** Foodborne outbreak contributing factors, National Environmental Assessment Reporting System, 2017–2019

	*n*	% of outbreaks^b^ (*n* = 393)
**Contamination of food with a foodborne illness agent** ^a^		
Toxic substance part of the tissue	12	3.1
Poisonous substance intentionally/deliberately added	0	0.0
Poisonous substance accidentally/inadvertently added	3	0.8
Addition of excessive quantities of ingredients that are toxic in large amounts	1	0.3
Toxic container	0	0.0
Contaminated raw product—food was intended to be consumed after a kill step	20	5.1
Contaminated raw product—food was intended to be consumed raw or undercooked/underprocessed	32	8.1
Foods originating from sources shown to be contaminated or polluted	2	0.5
Cross-contamination of ingredients	56	14.2
Bare-hand contact by a food worker who is suspected to be infectious	56	14.2
Glove-hand contact by a food worker who is suspected to be infectious	23	5.9
Other mode of contamination (excluding cross-contamination) by a food worker who is suspected to be infectious	96	24.4
Foods contaminated by non-food worker who is suspected to be infectious	8	2.0
Storage in contaminated environment	7	1.8
Other source of contamination	28	7.1
**Proliferation or growth of microbial agents in food (increase in number of bacteria or the production of toxins)** ^a^		
Food preparation practices that support proliferation of pathogens	25	6.4
No attempt was made to control the temperature of implicated food or the length of time food was out of temperature control	15	3.8
Improper adherence of approved plan to use time as a public health control	12	3.1
Improper cold holding due to malfunctioning refrigeration equipment	20	5.1
Improper cold holding due to an improper procedure or protocol	15	3.8
Improper hot holding due to malfunctioning equipment	1	0.3
Improper hot holding due to improper procedure or protocol	15	3.8
Improper/slow cooling	40	10.2
Prolonged cold storage	3	0.8
Inadequate modified atmosphere packaging	0	0.0
Inadequate processing	0	0.0
Other situations that promote or allow microbial growth or toxin production	6	1.5
**Survival of foodborne illness agents after a process, such as cooking, that should have eliminated or reduced them** ^a^		
Insufficient time and/or temperature during cooking/heat processing	24	6.1
Insufficient time and/or temperature during reheating	9	2.3
Insufficient time and/or temperature control during freezing	0	0.0
Insufficient or improper use of chemical processes designed for pathogen destruction	1	0.3
Other process failures that permit the pathogen to survive	7	1.8

a More than one contributing factor can be reported per outbreak.

b The denominator is the number of outbreaks that identified a contributing factor.

**Table 3.** Foodborne outbreak environmental antecedents, National Environmental Assessment Reporting System, 2017–2019

Antecedents^a^	n	% of antecedents^b^ (*n* = 1,430)	% of Outbreaks^c^ (*n* = 393)
**People (*n* = 964)**			
Lack of oversight of employees/enforcement of policies	350	24.5	89.1
Lack of training of employees on specific processes	291	20.3	74.0
Lack of a food safety culture/attitude towards food safety	226	15.8	57.5
Low/insufficient staffing	38	2.7	9.7
High turnover of employees or management	31	2.2	7.9
Language barrier between management and employees	28	2.0	7.1
**Equipment (*n* = 124)**			
Equipment is improperly used	35	2.4	8.9
Insufficient capacity of equipment	36	2.5	9.2
Poor facility layout	24	1.7	6.1
Lack of preventative maintenance on equipment	15	1.0	3.8
Improperly sized or installed equipment for the facility	14	1.0	3.6
**Economics (*n* = 85)**			
Lack of sick leave or other financial incentives to adhere to good practices	68	4.7	17.3
Lack of needed supplies for the operation of the restaurant	11	0.8	2.8
Lack of reinvestment in the restaurant	6	0.4	1.5
**Process (*n* = 192)**			
Employees or managers are not following the facility’s process	98	6.8	24.9
Insufficient process to mitigate the hazard	94	6.6	23.9
**Food (*n* = 18)**			
Food not treated as time and temperature control for safety	18	1.3	4.6
**Other (*n* = 47)**	47	3.3	12.0
**Total**	1430	100.0	– -

a Outbreaks could have more than 1 environmental antecedent.

b The denominator is the number of environmental antecedents reported for all outbreaks that reported at least one environmental antecedent.

c The denominator is the number of outbreaks that reported an environmental antecedent.

**Table 4:** Foodborne outbreak environmental antecedents by etiologic agent, National Environmental Assessment Reporting System, 2017–2019^a^

Antecedent	Norovirus		*Salmonella*		*Clostridium perfringens*	
	*n*	% of outbreaks^b^ (*n* = 156)	*n*	% of outbreaks^b^ (*n* = 53)	*n*	% of outbreaks^b^ (*n* = 25)
**People**						
Lack of training of employees on specific processes	80	51.3	29	54.7	14	56.0
Lack of oversight of employees/ enforcement of policies	123	78.8	32	60.4	12	48.0
High turnover of employees or management	7	4.5	4	7.5	0	0.0
Low/insufficient staffing	22	14.1	2	3.8	1	4.0
Lack of a food safety culture/ attitude towards food safety	61	39.1	28	52.8	9	36.0
Language barrier between management and employees	3	1.9	3	5.7	1	4.0
**Equipment**						
Insufficient capacity of equipment (not enough equipment for the processes)	5	3.2	4	7.5	7	28.0
Equipment is improperly used	2	1.3	5	9.4	3	12.0
Lack of preventative maintenance on equipment	1	0.6	4	7.5	1	4.0
Improperly sized or installed equipment for the facility	4	2.6	2	3.8	0	0.0
Poor facility layout	5	3.2	7	13.2	1	4.0
**Economics**						
Lack of reinvestment in the restaurant	0	0.0	2	3.8	2	8.0
Lack of sick leave or other financial incentives to adhere to good practices	41	26.3	5	9.4	1	4.0
Lack of needed supplies for the operation of the restaurant	4	2.6	1	1.9	1	4.0
**Process**						
Insufficient process to mitigate the hazard	13	8.3	12	22.6	9	36.0
Employees or managers are not following the facility’s process	41	26.3	12	22.6	5	20.0
**Food**						
Food not treated as time and temperature control for safety	0	0.0	3	5.7	2	8.0
**Other**	13	8.3	4	7.5	4	16.0
**Total**	425	–	159	–	73	–

a Outbreaks could have more than 1 agent and environmental antecedent.

b The denominator is the number of outbreaks that reported the agent (confirmed or suspected).

**Table 5:** Foodborne outbreak environmental antecedents by contributing factor, National Environmental Assessment Reporting System, 2017–2019^a^

	Other mode of contamination by suspected infectious worker		Cross-contamination of ingredients		Bare-hand contact by a suspected infectious worker	
	*n*	% of outbreaks^b^ (*n* = 96)	*n*	% of outbreaks^b^ (*n* = 56)	*n*	% of outbreaks^b^ (*n* = 56)
**People**						
Lack of training of employees on specific processes	49	51.0	35	62.5	37	66.1
Lack of oversight of employees/ enforcement of policies	76	79.2	40	71.4	43	76.8
High turnover of employees or management	3	3.1	5	8.9	4	7.1
Low/insufficient staffing	11	11.5	3	5.4	8	14.3
Lack of a food safety culture/attitude towards food safety	30	31.3	31	55.4	30	53.6
Language barrier between management and employees	6	6.3	4	7.1	1	1.8
**Equipment**						
Insufficient capacity of equipment (not enough equipment for the processes)	2	2.1	6	10.7	2	3.6
Equipment is improperly used	1	1.0	5	8.9	1	1.8
Lack of preventative maintenance on equipment	0	0.0	2	3.6	1	1.8
Improperly sized or installed equipment for the facility	1	1.0	1	1.8	2	3.6
Poor facility layout	4	4.2	7	12.5	1	1.8
**Economics**						
Lack of reinvestment in the restaurant	0	0.0	2	3.6	0	0.0
Lack of sick leave or other financial incentives to adhere to good practices	29	30.2	3	5.4	14	25.0
Lack of needed supplies for the operation of the restaurant	0	0.0	1	1.8	3	5.4
**Process**						
Insufficient process to mitigate the hazard	6	6.3	7	12.5	6	10.7
Employees or managers are not following the facility’s process	30	31.3	11	19.6	10	17.9
**Food**						
Food not treated as time and temperature control for safety	0	0.0	1	1.8	0	0.0
**Other**	6	6.3	1	1.8	3	5.4
**Total**						

a Outbreaks could have more than 1 contributing factor and environmental antecedent.

**Table 6:** Outbreak etiologic agents, contributing factors, and environmental antecedents by outbreak year, National Environmental Assessment Reporting System, 2017–2019^a^

	2017 (*n* = 88)		2018 (*n* = 152)		2019 (*n* = 153)	
Etiologic agents^b^	n	%	n	%	n	%
Norovirus	42	54.5	58	48.7	56	48.7
*Salmonella*	12	15.6	21	17.6	20	17.4
*Clostridium perfringens*	7	9.1	10	8.4	8	7.0
**Contributing factors** ^c^						
Other contamination by a suspected infectious worker	29	33.0	34	22.4	33	21.6
Cross-contamination of ingredients	11	12.5	23	15.1	22	14.4
Bare-hand contact by a suspected infectious worker	18	20.5	21	13.8	17	11.1
**Environmental antecedents** ^c^						
Lack of employee oversight/enforcement of policies	75	85.2	124	81.6	151	98.7
Lack of employee training	64	72.7	111	73.0	116	75.8
Lack of a food safety culture/attitude towards food safety	55	62.5	83	54.6	88	57.5

a Outbreaks could have more than 1 agent, contributing factor, and environmental antecedent.

b The denominator is the number of outbreaks that reported an agent (confirmed or suspected). 2017: *n* = 77; 2018: *n* = 119; 2019: *n* = 115.

c The denominator is the number of outbreaks that reported a contributing factor and environmental antecedent. 2017: *n* = 88; 2018: *n* = 152; 2019: *n* = 153.
